# Retraction: Frequencies of *Porphyromonas gingivalis* detection in oral-digestive tract tumors

**DOI:** 10.3389/pore.2023.1611616

**Published:** 2023-12-19

**Authors:** Pathology and Oncology Research Editorial Office

**Affiliations:** Frontiers Media SA, Lausanne, Switzerland

The journal retracts the 1 April 2021 article cited above.

Following publication, concerns were raised regarding data misrepresentation. Following provision of raw data by the authors, the Editor in Chief concluded that the article’s conclusions and assertions were not sufficiently supported by the findings from the material provided; therefore, the article has been retracted. The authors do not agree to this retraction.

